# Survival outcomes following total intravenous versus volatile anesthesia in esophagogastric cancer surgery: a systematic review and meta-analysis

**DOI:** 10.1007/s00423-026-04329-3

**Published:** 2026-10-07

**Authors:** Efstathia Liatsou, Alexandra Pliakopanou, Dimitrios Liatsos, Elisabeth Nagy, Ioannis Karniadakis, Kaveh Khodakaram, Dimitrios Schizas, Ioannis Rouvelas

**Affiliations:** 1 Department of Surgery, Sahlgrenska University Hospital, Region Västra Götaland, Gothenburg, Sweden; 2 Department of Medicine, School of Health Sciences, University of Ioannina, Ioannina, Greece; 3 Department of Medicine, National and Kapodistrian University of Athens, Athens, Greece; 4 Center for Advanced Medical Simulation and Training (CAMST), Karolinska University Hospital, Stockholm, Sweden; 5 Clinical Sciences, Intervention and Technology (CLINTEC), Karolinska Institute, Stockholm, Sweden; 6 Queen Elizabeth Hospital, Lewisham and Greenwich NHS Trust, London, UK; 7 First Department of Surgery, National and Kapodistrian University of Athens, Laikon General Hospital, Athens, Greece; 8 Department of Upper Abdominal Surgery, Center for Digestive Diseases, Karolinska University Hospital, Huddinge, Stockholm Sweden; 9 Division of Surgery and Oncology, Department of Clinical Science, Intervention and Technology (CLINTEC), Karolinska Institutet, Huddinge, Stockholm Sweden

**Keywords:** Esophagogastric cancer, Total intravenous anesthesia, Volatile anesthesia, Overall survival, Recurrence-free survival, Meta-analysis

## Abstract

**Purpose:**

Esophagogastric cancers remain among the leading causes of cancer-related mortality worldwide. Surgical resection represents the primary curative treatment strategy; however, perioperative factors may influence long-term oncological outcomes. Experimental and clinical evidence suggests that anesthetic techniques may affect tumor progression and recurrence through immunological and oncological pathways. Total intravenous anesthesia (TIVA) has been proposed to exert immunomodulatory and antitumor effects compared to volatile anesthesia (VA), although evidence remains inconclusive. This systematic review and meta-analysis aimed to evaluate the impact of TIVA versus volatile anesthesia on survival outcomes in patients undergoing esophagogastric cancer surgery.

**Methods:**

A systematic review and meta-analysis was conducted according to PRISMA 2020 guidelines. PubMed and Scopus were searched for studies published between 2010 and 2025 comparing TIVA with volatile anesthesia in adult patients undergoing curative surgery for esophageal or gastric cancer. The primary outcome was overall survival (OS). Secondary outcomes included recurrence-free survival (RFS), early mortality, operation time, and postoperative hospital stay. Pooled hazard ratios (HR), risk ratios (RR), and mean differences (MD) with 95% confidence intervals (CI) were calculated using random-effects models.

**Results:**

Thirteen studies (3 randomized controlled trials and 10 retrospective studies) involving 264,543 patients were included. Of these, 41,977 patients received TIVA and 222,566 received volatile anesthesia. Pooled analysis of eight studies demonstrated significantly improved overall survival with TIVA (HR 0.80, 95% CI 0.69–0.93; *p* = 0.003). Subgroup analysis showed a significant survival benefit in patients undergoing gastrectomy (HR 0.74, 95% CI 0.60–0.91; *p* < 0.004), whereas no significant difference was observed for esophagectomy (HR 0.89, 95% CI 0.73–1.10; *p* = 0.29). TIVA was associated with non-significant trends toward improved recurrence-free survival (HR 0.91, 95% CI 0.77–1.06; *p* = 0.22) and reduced early mortality (HR 0.90, 95% CI 0.77–1.05; *p* = 0.17). No statistically significant differences were observed in operation time (MD − 3.81 min, 95% CI − 10.75 to 3.13) or hospital stay (MD − 0.19 days, 95% CI − 0.39 to 0.00; *p* = 0.06).

**Conclusions:**

TIVA may be associated with improved long-term survival outcomes compared with volatile anesthesia in patients undergoing esophagogastric cancer surgery, particularly among patients undergoing gastrectomy. Given the predominance of retrospective evidence and methodological heterogeneity, further high-quality prospective randomized studies are required to clarify the oncological impact of anesthetic techniques in upper gastrointestinal cancer surgery.

**Supplementary Information:**

The online version contains supplementary material available at https://doi.org/10.1007/s00423-026-04329-3.

## Introduction

Esophageal and gastric cancers remain a global health concern, ranking as the eleventh and fifth most common malignancies and as the seventh and fifth leading causes of cancer-related mortality, respectively, both characterized by a poor long-term prognosis [1]. Despite recent advances, with novel chemotherapeutic and immunotherapeutic agents entering the treatment regimen, surgical resection remains the mainstay of curative treatment, particularly in non-metastatic disease [2, 3]. Despite its vital role in cancer treatment, surgery itself may contribute to processes involved in local recurrence and distant metastases. Theories behind this phenomenon center on the concepts of cancer cell dissemination into the systemic circulation, destruction of local vasculature, and stress-induced cell-mediated immunosuppression [4, 5]. Alongside surgery, countless other parameters seem to be involved in the perioperative course of the tumor, including the type of anesthesia that is going to be implemented [6].

Both esophageal and gastric surgery require general anesthesia in order to be performed, which can be induced either by total intravenous anesthesia (TIVA), using agents such as propofol, or by volatile anesthesia (VA), using agents such as sevoflurane, isoflurane, and desflurane. No consensus has been reached regarding a preference between the two methods, but recent studies are beginning to unveil differences in their effects on the immune system [6, 7]. Most notably, VA has been suggested to have a suppressive effect on both innate and adaptive immunity, thus predisposing patients to tumor recurrence, growth, and metastases. Induction of cancer cell proliferation, apoptosis, and invasion, while natural killer cells and lymphocytes are suppressed, seems to be behind this effect. On the other hand, TIVA, specifically propofol-based regimens, has been suggested to have immunomodulatory and antitumor effects, such as reducing tumor survivability, progression, and invasion of cancer cells, while also activating and mobilizing T-lymphocytes, which play a key role in antitumor response [8, 9]. These different effects on the immune system and thus the antitumor response have been the basis for various studies, which seek to translate these differences into survival advantages of TIVA over VA. Several of these studies have been conducted in esophageal and gastric cancer surgery. Although some studies have shown the superiority of TIVA in terms of survival and oncologic results, there is a lack of a final verdict, as the results either contradict themselves or lack statistical significance [10]. To date, evidence specifically addressing the impact of anesthetic technique in esophagogastric cancer surgery remains limited and heterogeneous.

This meta-analysis aims to synthesize the available evidence and clarify the effect of different anesthetic agents on survival in esophagogastric oncologic surgery.

## Methods

### Study design

The present systematic review and meta-analysis was designed in accordance with the Preferred Reporting Items for Systematic Reviews and Meta-Analyses (PRISMA, 2020) guidelines [11]. As this study was based exclusively on previously published data, neither patient consent nor Institutional Review Board approval was required. The study was registered in Prospero (registration number: CRD420261428372).

### Search strategy

A comprehensive literature search was conducted in PubMed and Scopus for studies published from 2010 to 2025. The search strategy was adapted to the indexing system and search interface of each database, consisting of both controlled vocabulary (MeSH terms) and free-text terms. The PubMed search strategy was as follows: (“Neoplasms/surgery”[MeSH Terms]) AND (“Anesthesia, Intravenous”[MeSH Terms] OR “Anesthesia, Inhalation”[MeSH Terms]) AND (survival OR outcomes OR mortality). For Scopus, the search strategy was adapted to: TITLE-ABS-KEY (cancer AND surgery AND (intravenous anesthesia OR inhalation anesthesia) AND (survival OR mortality OR outcomes)). Reference list checking (backward citation searching) and forward citation searching (snowballing) were performed in addition to database searching to identify eligible articles. The final search was conducted on March 30, 2026.

### Eligibility criteria

Studies were selected according to predefined eligibility criteria based on the PICO framework. The population of interest included adult patients with gastric or esophageal cancer undergoing curative surgical resection. The intervention was total intravenous anesthesia, compared with inhalational anesthesia during the maintenance phase. Induction anesthesia with intravenous agents was accepted in both arms. Eligible studies were required to report at least one clinically relevant outcome, including survival and mortality. Eligible study designs included randomized controlled trials (RCTs) and observational (prospective and retrospective) cohort studies. Only studies published between 2010 and 2025 were considered. Studies involving surgery for non-malignant gastric or esophageal diseases, non-gastroesophageal malignancies, pediatric populations, case reports, reviews, editorials, and preclinical studies (animal and in vitro) were excluded. Studies not reporting outcomes of interest were also excluded.

### Study selection and data extraction

Title and abstract screening of studies identified through the systematic literature search were followed by full-text evaluation against the inclusion criteria. Disagreements at any stage of the selection process were resolved through discussion. An electronic extraction spreadsheet was created, pilot-tested, and adjusted accordingly after discussion within the study group. To minimize bias, data extraction was performed independently and in a blinded manner by two reviewers. Where consensus was not reached, a third independent reviewer adjudicated.

Data extraction included: (a) study characteristics (name of first author, publication year, PubMed ID, study design, study center, country, and continent), (b) patient characteristics (total sample size, sex, age, body mass index, American Society of Anesthesiologists (ASA) status, comorbidities), (c) tumor characteristics (type of cancer, tumor location, histopathological type, and pathological stage based on the TNM classification), (d) perioperative variables (type of anesthesia (total intravenous anesthesia and volatile anesthesia, with corresponding number of patients and agents used), use of epidural anesthesia, type of surgery, neoadjuvant and adjuvant therapy, duration of surgery and anesthesia, and resection margin status).

**Fig. 1:**
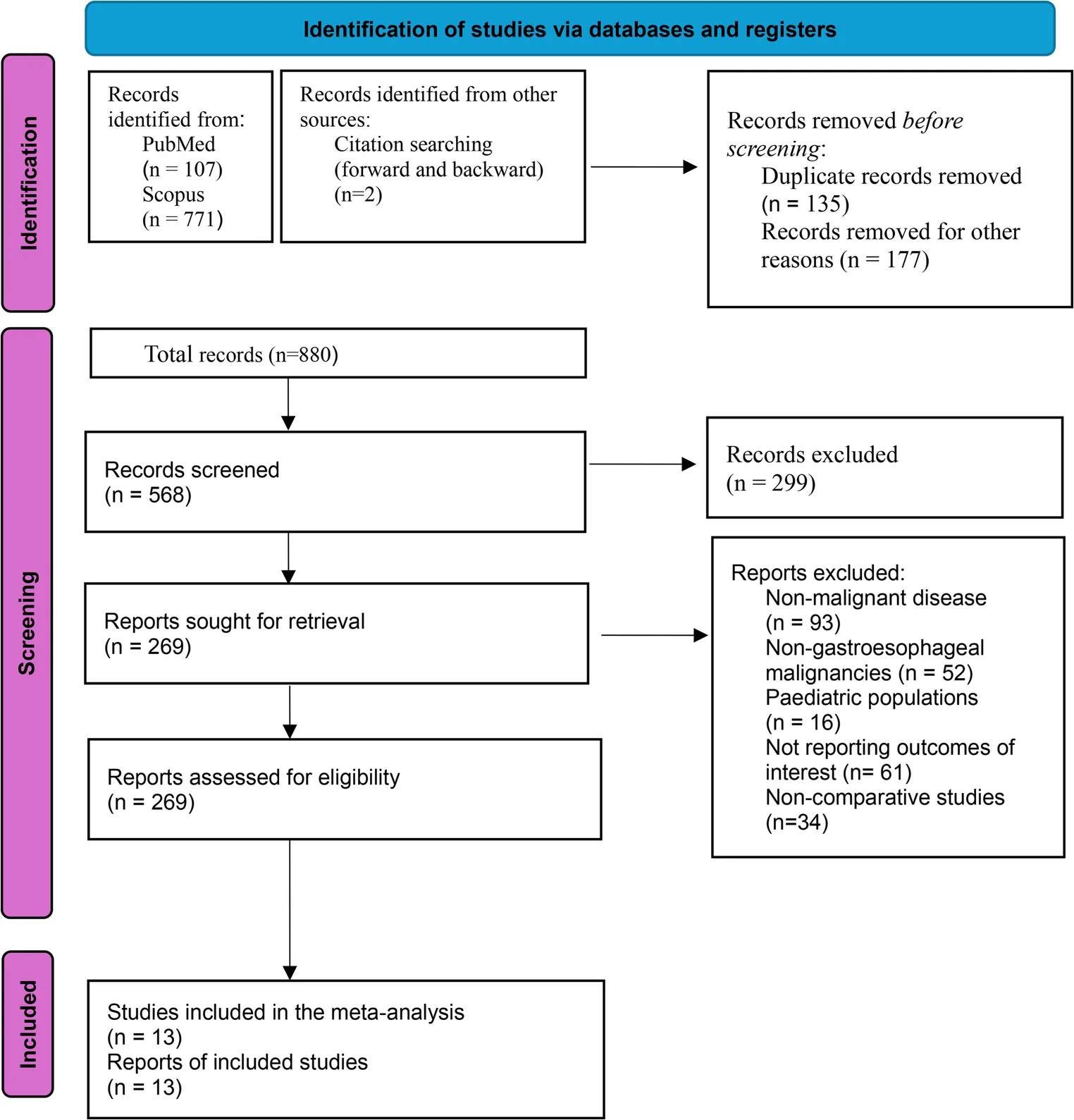
PRISMA flow chart for screening and inclusion of studies, eligibility assessment, and inclusion of studies in the systematic review and meta-analysis

The primary outcome was overall survival, while secondary outcomes included recurrence-free survival (RFS), early mortality, length of postoperative hospital stay, and operation time. When necessary, effect estimates were transformed into a common metric for analysis. Continuous variables were extracted as means and standard deviations. The PRISMA flow chart describing the screening and selection process of included studies is presented in Fig. 1.

### Quality assessment

Risk of bias and methodological quality of the included studies were assessed independently by two reviewers. Randomized controlled trials were evaluated with the Cochrane Risk of Bias 2 (RoB 2) tool [12], which assesses bias arising from the randomization process, deviations from intended interventions, missing outcome data, measurement of outcomes, and selection of reported results. Observational studies were evaluated with the ROBINS-I tool [13], which assesses bias due to confounding, selection of participants, classification of interventions, deviations from interventions, missing data, measurement of outcomes, and selection of reported results.

Each study was assigned an overall risk of bias based on the respective tools: low risk, some concerns, or high risk for RoB2, and low, moderate, serious, or critical risk for ROBINS-I. Any disagreements were resolved through discussion and consensus.

### Statistical analysis

A meta-analysis was performed using RevMan 5.4. (The Cochrane Collaboration, Copenhagen, Denmark, version 2016). Dichotomous variables were assessed using the risk ratio (RR), continuous variables were assessed using the mean difference, and survival was assessed using the hazard ratio (HR). Heterogeneity was considered statistically significant at *p* < 0.10 on Cochran’s Q test, and the degree of inconsistency was quantified using the I² statistic. The 95% confidence intervals (CI) were reported for all results. A random-effects model was used for the pooled results. Publication bias was assessed with funnel plots and Egger’s test. (Supplementary Figs. 3–10)

When mean values and standard deviations were not reported in the studies, values were calculated according to the equations proposed by Hozo et al. [14] The predetermined level of statistical significance for the outcomes studied in this meta-analysis was set at *p* < 0.05.

Hazard ratios obtained from univariate and multivariate analyses were analyzed separately because these estimates represent different statistical constructs. Univariate HRs reflect crude associations, whereas multivariate HRs account for potential confounding variables and provide adjusted effect estimates. However, several studies did not mention which variables were adjusted for in the multivariate analysis. Therefore, separate meta-analyses were performed to preserve comparability of effect estimates and improve interpretability of pooled results.

## Results

Overall, 13 studies (3 RCTs and 10 retrospective studies) were included in the qualitative and quantitative analysis with a total of 264,543 patients, of whom 77,764 (68.16%) were male and 36,333 (31.84%) were female [15–27]. Sex-specific data were not available in all included studies. A total of 41,977 (15.87%) patients received TIVA, and 222,566 (84.13%) received inhaled anesthetics. From studies with available demographic data (total: 111,241 patients), the median age was 63.09 years (SD: 11.72 years). Nine studies had results from patients who underwent gastrectomy, whereas outcomes from patients who underwent esophagectomy were available from 7 studies. Of studies with available data, 727 (19.36%) patients who received TIVA had ASA score I, 2,720 (72.38%) had ASA score II, and 318 (8.26%) had ASA score III. Of patients who received volatile anesthetic agents, 2,015 (31.84%) had ASA score I, 3,756 (59.36%) had ASA score II, and 557 (8.8%) had ASA score III. In the TIVA group, 1,415 (38.03%) patients had TNM stage I, 1,238 (33.27%) had stage II, 1,039 (27.92%) had stage III, and 29 (1%) had stage IV. In the volatile group, 1,238 (26.27%) patients had TNM stage I, 1,463 (31.04%) patients had stage II, 1,969 (41.78%) had stage III, and 43 (1%) had stage IV. Among studies with available data, minimally invasive surgery was performed in 1,024 patients in the TIVA group and 3,203 patients in the volatile group. Regarding comorbidities, in the TIVA group, 1,800 (11.19%) patients had chronic pulmonary disease, 1,185 (7.37%) were active smokers, 2,373 (14.75%) had coronary artery disease, 963 (6%) had cerebrovascular disease, 4,595 (28,56%) had hypertension, and 5,172 (32.13%) had diabetes mellitus (DM). In the volatile group, 14,403 (9.59%) had chronic pulmonary disease, 1,080 (1%) were active smokers, 20,899 (13.91%) had coronary artery disease, 10,345 (6.9%) had cerebrovascular disease, 47,122 (31.36%) had hypertension, 3,704 (2.5%) had liver disease, and 52,706 (34.74%) had DM.

### Survival outcomes

**Fig. 2:**
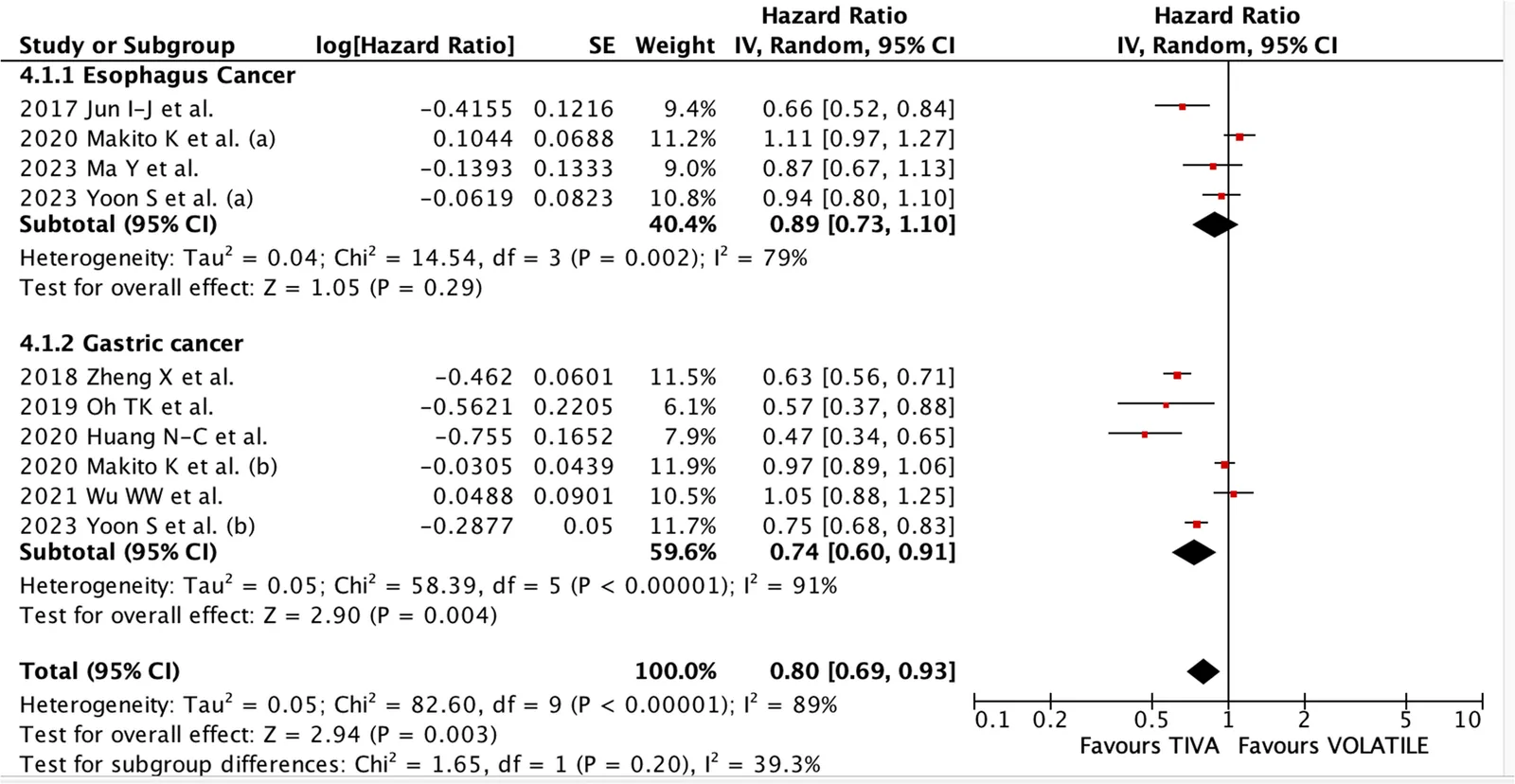
Pooled analysis for overall survival in studies including patients undergoing esophageal and gastric cancer surgery

Figure 2 presents the pooled analysis of 8 studies with available outcomes on overall survival for esophageal cancer and gastric cancer surgery. Values of HR < 1 indicate point estimates in the direction of TIVA in comparison with volatile anesthetics; however, statistical significance varied by surgical site. The pooled HR for esophagectomy was 0.89 (95% CI 0.73–1.10; *p* = 0.290), and for gastrectomy, 0.74 (95% CI 0.60–0.91; *p* = 0.004), favoring the use of TIVA. The pooled overall HR across all included studies was 0.80 (95% C.I. 0.69–0.93; *p* = 0.003) and was statistically significant.

**Fig. 3:**
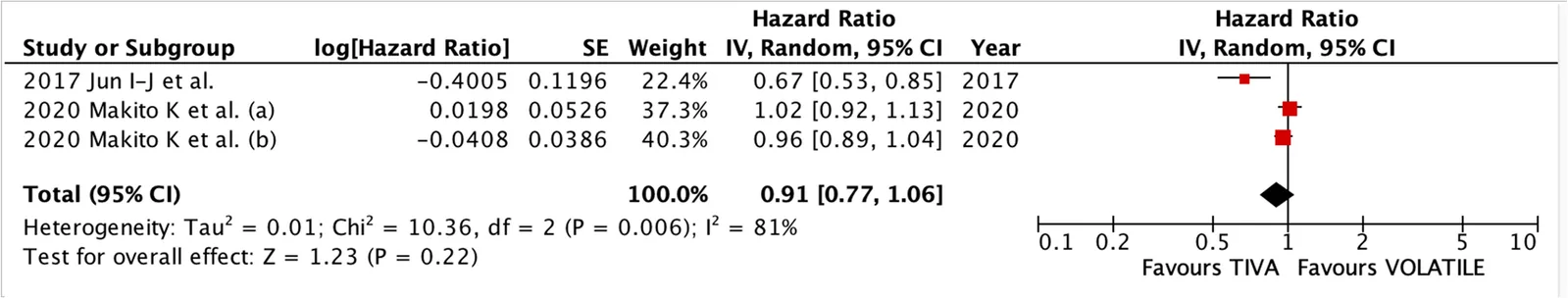
Pooled analysis of recurrence-free survival in studies including patients undergoing upper gastrointestinal cancer surgery

**Fig. 4:**
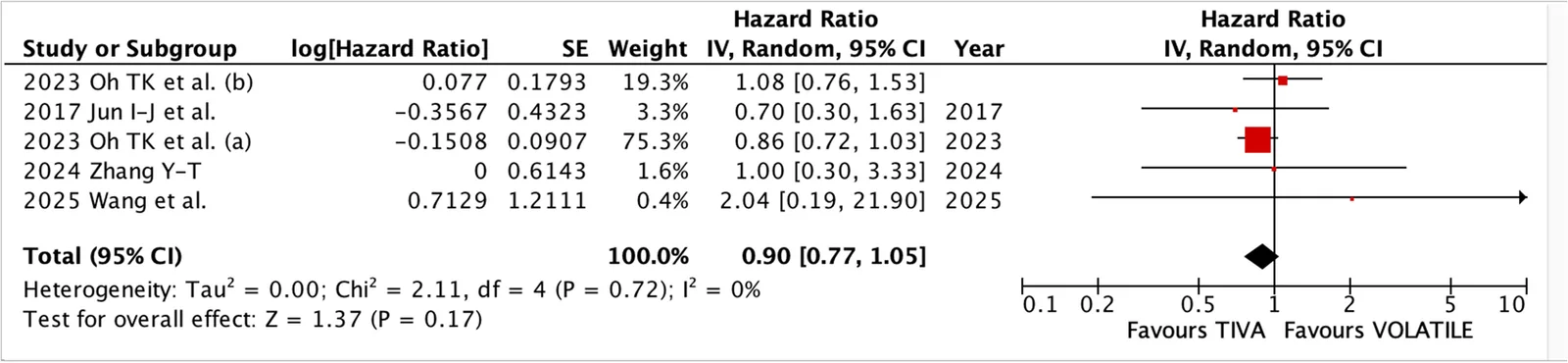
Pooled analysis of early mortality in patients undergoing upper gastrointestinal cancer surgery

**Fig. 5:**
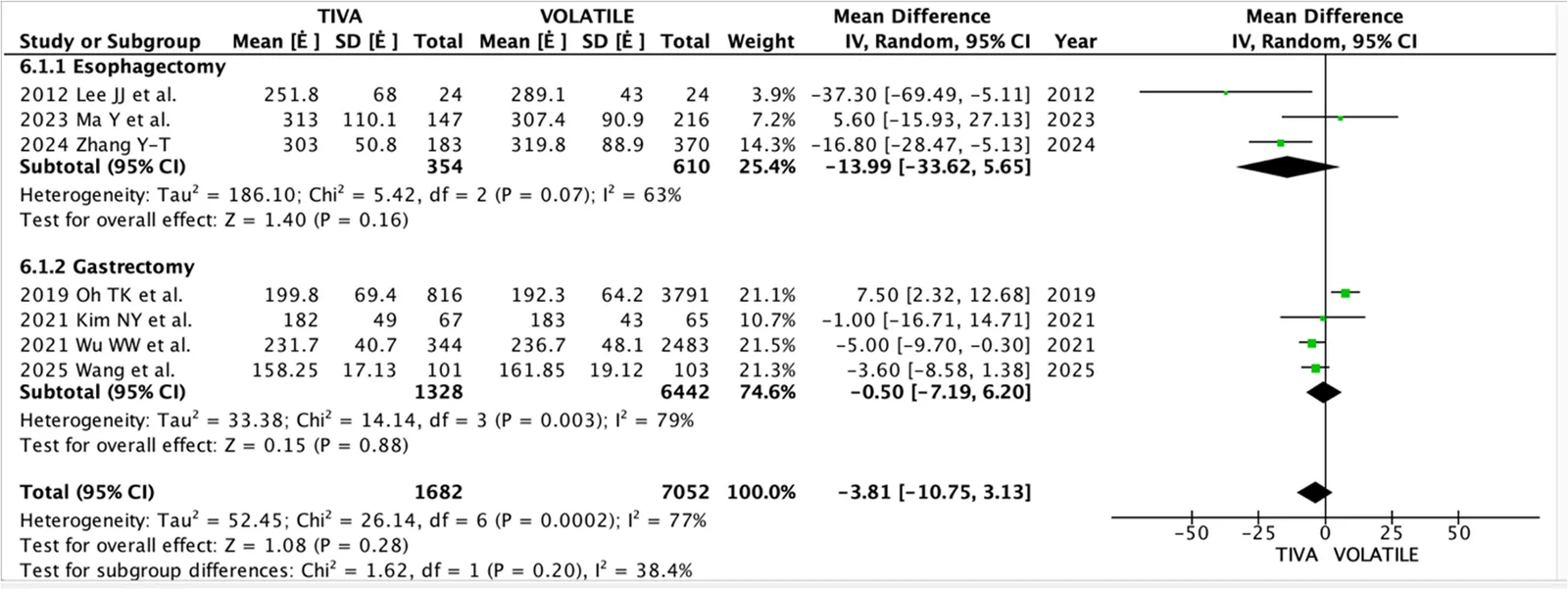
Pooled mean difference analysis of operative time between the TIVA and volatile anesthesia groups in patients undergoing upper gastrointestinal cancer surgery

**Fig. 6:**
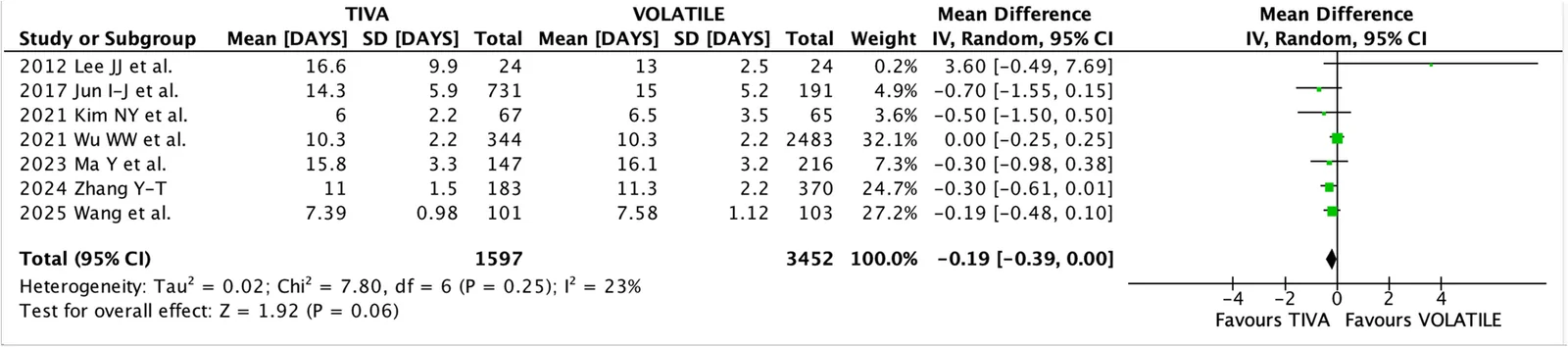
Pooled mean difference analysis of postoperative hospital stay between the TIVA and volatile anesthesia groups in patients undergoing upper gastrointestinal cancer surgery

In secondary outcomes, point estimates for RFS, early mortality, hospital stay, and operation time were in the direction of TIVA; however, none reached statistical significance. More specifically, the pooled HR for RFS from 3 studies was 0.91 (95% CI 0.77–1.06; *p* = 0.22), and for early mortality was 0.90 (95% CI 0.77–1.05; *p* = 0.17, favoring TIVA (Figs. 3 and 4, respectively). A pooled mean difference analysis was performed to compare the operation time between the two groups. No statistically significant difference in operation time was found between the two groups, with a mean difference of -3.81 min (95% CI -10.75 to 3.13), *p* = 0.20 (Fig. 5). No statistical significance was shown in the hospital stay between the two groups, with a mean difference of -0.19 (95% CI -0.39 to 0.00) days, *p* = 0.06 (Fig. 6). The heterogeneity and the pooled results of studies for each outcome are presented in the forest plots.

**Fig. 7:**
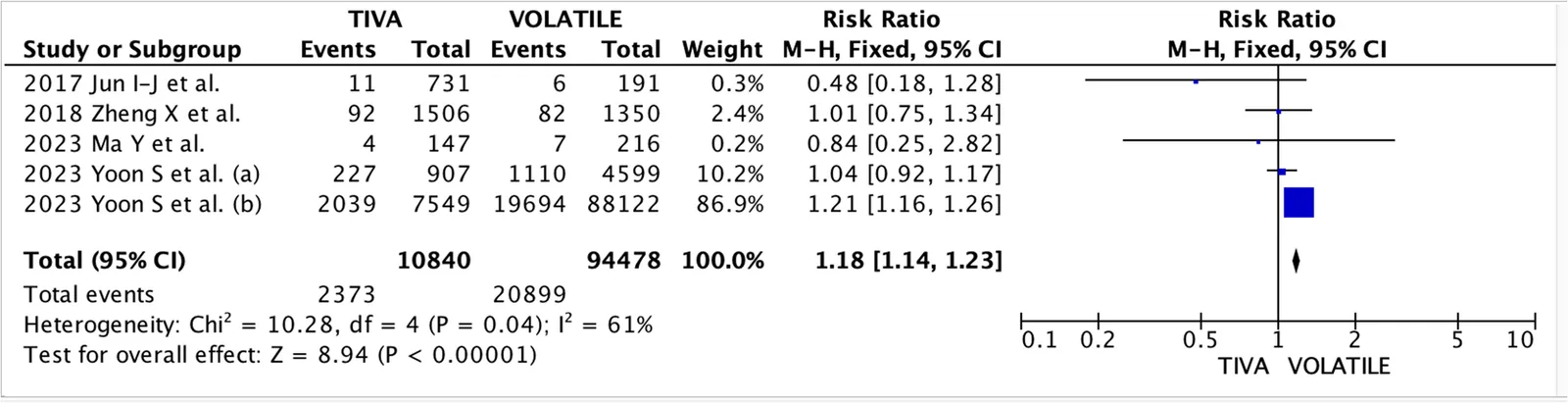
Pooled proportional meta-analysis of patients with coronary artery disease receiving either TIVA or volatile anesthesia

**Fig. 8:**
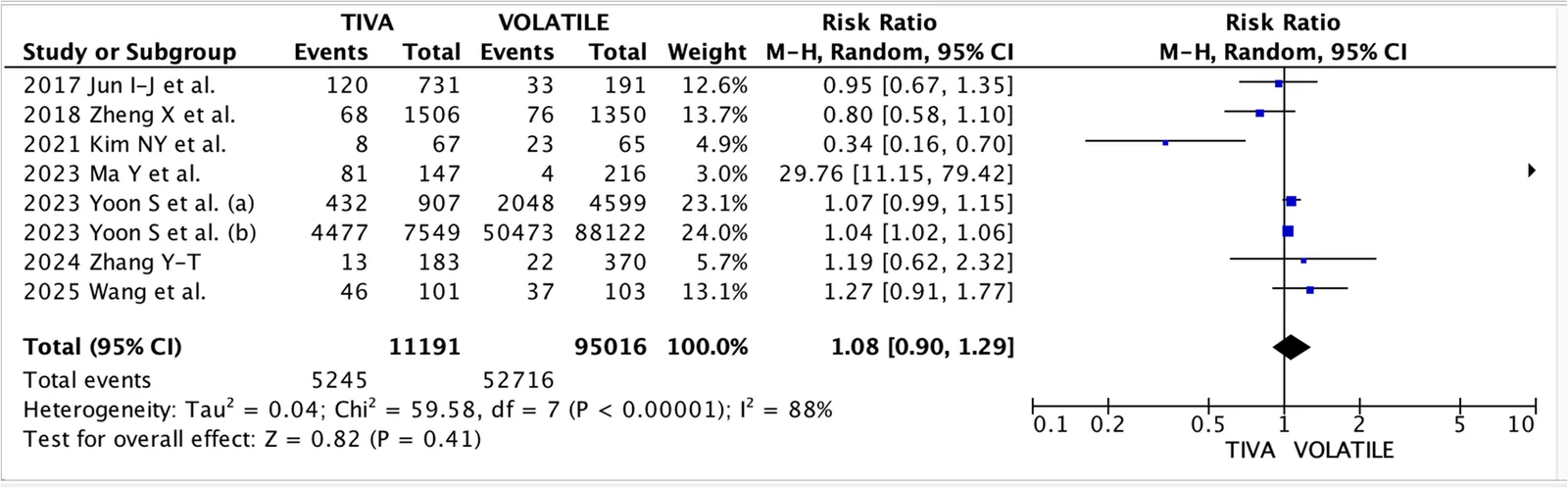
Pooled proportional meta-analysis of patients with diabetes mellitus receiving either TIVA or volatile anesthesia

Patients with coronary artery disease received volatile anesthesia more frequently than TIVA (RR: 1.18 (95% CI 1.14–1.23; *p* < 0.00001), whereas no statistically significant difference was observed for diabetes mellitus (DM) (RR: 1.08, 95% CI 0.90–1.29; *p* = 0.41) (Figs. 7 and 8).

The risk of bias assessment of the studies included is shown in Supplementary Figs. 1 and 2. Among the RCTs, most studies were judged as having low risk of bias or some concerns, whereas one study was considered as high risk of bias. Across domains, the majority of studies demonstrated low risk in randomization, missing outcome data, outcome measurement, and selective reporting. In retrospective studies, overall methodological quality varied substantially across studies. Most studies were judged to have moderate-to-serious risk of bias, primarily due to confounding and participant selection. Several studies were classified as having serious risk of bias, particularly in the domains of confounding and selection bias. Few studies achieved low overall risk of bias.

## Discussion

The primary finding of this study was that TIVA was associated with significantly improved overall survival, while non-significant trends towards improved recurrence-free survival and lower early mortality were observed in comparison with volatile anesthetics in patients undergoing esophagogastric cancer surgery. The subgroup analysis showed that studies with gastrectomy represented 59.6% of the overall weight, and the statistically significant survival benefit was primarily driven by this subgroup. No statistically significant differences were observed in the operation time and length of hospital stay between the two agents, while the mechanisms underlying the observed survival association cannot be established from the present clinical data. We also found that patients with coronary artery disease were more likely to receive volatile anesthesia, whereas no significant difference was observed for DM. These differences may reflect baseline differences in patient selection between the two groups.

An interesting finding was that the survival benefit associated with TIVA appeared to be driven primarily by patients undergoing gastrectomy, whereas no statistically significant difference was observed among patients undergoing esophagectomy. Several explanations may account for this observation. First, the gastrectomy subgroup contributed a larger proportion of the overall study weight, providing greater statistical power to detect differences between anesthetic techniques. Importantly, the direction of effect in the esophagectomy subgroup also showed a point estimate in the direction of TIVA (HR 0.89), suggesting that the absence of statistical significance should not necessarily be interpreted as evidence of no effect. Second, gastric cancer is characterized by a higher propensity for peritoneal dissemination and systemic recurrence [28], processes that could potentially be influenced by perioperative immune modulation. In contrast, outcomes following esophagectomy are heavily influenced by factors such as pulmonary complications, anastomotic leaks, cardiopulmonary comorbidity, and one-lung ventilation, which may dilute any potential oncological effect of anesthetic technique [29, 30]. Furthermore, although esophagectomy and gastrectomy are frequently grouped as upper gastrointestinal cancer surgery, they represent substantially different procedures with respect to surgical trauma, perioperative inflammatory response, cardiopulmonary stress, and postoperative morbidity [31]. These differences may influence the relative contribution of anesthetic technique to long-term outcomes and support the notion that gastric and esophageal cancer surgery should not necessarily be considered a homogeneous population when evaluating oncological effects of anesthesia. Future studies should therefore evaluate gastric and esophageal cancer separately and be adequately powered for tumor-specific subgroup analyses.

Scientific interest in the effect of intraoperative anesthesia on long-term oncological outcomes through systemic immunological mechanisms has been gaining increasing impact, especially in digestive cancer and thoracic surgery [19, 32]. No statistical differences in overall survival were shown between patients who received TIVA and volatile anesthesia for gastrointestinal surgery; however, total intravenous anesthesia was found to be associated with better recurrence-free survival [19]. Subgroup analysis did not reveal any confounding factors that could affect the survival outcomes. A meta-analysis by Beverstock et al. analyzed the mortality of patients who underwent cardiac surgery and received either total intravenous or volatile anesthesia and did not show any statistically significant differences in both 30-day and 1-year mortality [32]. Beneficial effects on cardioprotection with volatile anesthetics were shown in a meta-analysis of 27 trials involving patients who underwent coronary artery bypass grafting (CABG) surgery. Although differences in hard clinical outcomes such as myocardial ischemia (MI) and hospital mortality were not affected by the type of anesthesia, there was a statistically significant impact on biochemical values [33]. Patients randomized to receive volatile anesthesia had 20% higher cardiac indices, significantly lower serum troponin I concentrations, shorter duration of mechanical ventilation, and a shorter length of hospital stay [33]. Despite the well-known cardioprotective role of volatile anesthetics, this study did not yield conclusive or generalizable results due to differences in administered doses, ventilatory management protocols, and the use of cardiac arrhythmia-inducing inotropic agents.

The difficulty in drawing unambiguous conclusions regarding the effect of anesthetic agents on cancer surgery stems from the fact that both volatile and TIVA present with potentially conflicting cardiopulmonary and immuno-oncological effects. Volatile anesthetics mimic ischemic preconditioning by activating mitochondrial potassium ATP channels that regulate myocardial recovery and trigger protective signaling pathways [34]. Preclinical and clinical studies show that inhaled anesthetics may both prevent and reduce inflammatory lung injury by decreasing the release of pro-inflammatory cytokines such as IL-6, IL-8, and NF-kB [35]. Bronchoalveolar lavage fluid from patients undergoing thoracic surgery with one-lung ventilation (OLV) showed significantly lower inflammatory markers in the ventilated lung, suggesting a local anti-inflammatory function [36]. This finding has been proposed as one potential explanation for the reduced mortality observed in some cardiac surgery studies; however, the potential benefit in reducing pulmonary and other complications was not profound in non-cardiac surgeries, possibly due to heterogeneity and underrepresentation of these surgeries [37]. 

Those immunomodulatory and cytoprotective features have been shown to have contradictory molecular actions depending on the type of cancer. For example, in colon cancer, sevoflurane is suggested to inhibit proliferation and invasion through inhibition of the Ras/Raf/MEK/ERK signaling pathway [38]. Opposite results occur in gynecological cancers, where sevoflurane has been associated with enhanced cell proliferation, migration, and invasion in cervical cancer cell cultures via induction of histone deacetylase 6 expression via the ERK1/2 and phosphatidylinositide 3-kinase/AKT signaling pathways [39]. Propofol has been demonstrated to possess a range of antitumor properties by directly regulating key signaling pathways in tumorigenesis, such as the MAPK and NF-kB pathways, as well as regulating the expression of miRNA and HIF-1a [40]. Potential tumor-related effects of anesthetic agents have also been described in gastric cancer models, where incubation with sevoflurane was found to induce apoptosis through inhibiting WIF-1 expression and upregulating β-catenin expression [41]. The scientific hypothesis that surgical stress can produce pro-inflammatory mediators that favor tumor growth and metastasis has driven increasing interest in examining intraoperative modalities, including anesthesia. Τhus, clinical data from prospective studies remain underrepresented. A large prospective randomized study has been assessed the oncological impact of intraoperative anesthetics administered to patients undergoing breast cancer surgery [42]. Patients were randomly allocated to receive either regional paravertebral anesthesia in combination with propofol-based (TIVA) or sevoflurane inhalational anesthesia plus opioid analgesia.

Surgery for gastroesophageal cancer is a complicated procedure where postoperative survival can be affected by both cardiopulmonary parameters, postoperative complications, and the oncological outcome of the resection. In an RCT, Kim et al., investigated the effect of anesthetics on perioperative changes in syndecan-1, a proteoglycan found on the endothelial glycocalyx (EG), that coats the luminal surface of the vascular endothelium and depicts the grade of EG breakdown during stress-inducing procedures [22]. Serum syndecan-1 concentrations increased significantly from preoperative levels in the volatile anesthesia group, whereas, the TIVA group showed significantly lower levels of syndecan-1 by the end of surgery (23.4 ± 11.7 vs. 29.3 ± 14.7 ng/mL; Bonferroni-corrected *p* = 0.021). This finding correlated with laboratory values, including WBC count and neutrophil-to-lymphocyte ratio [22]. Most relevant evidence regarding upper gastrointestinal surgery derives from bariatric surgery studies. A systematic review by Kamal et al. showed that TIVA was associated with a lower incidence of postoperative nausea and vomiting, while other results focused on intraoperative vital signs, time to emerge from anesthesia, length of postoperative intensive care unit stay, and rates of ICU admissions [43]. These findings suggest that anesthetic techniques may differentially influence perioperative physiological and inflammatory responses; however, whether such effects contribute to long-term oncological outcomes remains uncertain [43]. 

This is the first systematic review and meta-analysis comparing the use of ΤΙVA and volatile anesthetics in esophagogastric surgery, in contrast to previous studies that included both cardiac and non-cardiac surgery. Our study demonstrated a statistically significant association between TIVA and improved overall survival, particularly among patients undergoing gastrectomy. We also observed that patients with coronary artery disease more frequently received volatile anesthesia, possibly reflecting baseline differences between the treatment groups. However, our study has several limitations. First, the analysis included 10 retrospective studies, most of which were assessed as having a moderate-to-serious risk of bias. Although several studies reported adjusted estimates, the variables included in the multivariable models differed between studies, and residual confounding cannot be excluded. In addition, the studies covered a one-decade period (2012–2025), during which advances in perioperative care and surgical techniques may have influenced the outcomes. Regarding the type of surgery and the use of minimally invasive techniques, data on the specific anesthetic approach used in each case were unavailable, making subgroup analyses for these important confounding factors impossible. With respect to early mortality, the study by Oh TK accounted for 75.3% of the total patient population included in the analysis, thereby heavily influencing the results. A sensitivity analysis excluding this study was therefore performed, and he point estimate remained in the direction of TIVA, although the result was not statistically significant (HR 0.86, 95% CI: 0.44–1.70). (Supplementary Fig. 11). Finally, data regarding disease stage and patient comorbidities were not consistently available, which may have influenced the choice of anesthetic technique and introduced residual confounding. Heterogeneity was also observed across retrospective studies with respect to induction and maintenance protocols, as bolus doses of propofol were occasionally used even when volatile anesthesia constituted the primary maintenance technique. In addition, although PubMed/MEDLINE and Scopus provided broad and complementary literature coverage, the use of only two electronic databases may have resulted in relevant studies being missed.

Furthermore, variations in perioperative care pathways and institutional management protocols may have contributed to additional heterogeneity across studies.

## Conclusions

This systematic review and meta-analysis suggests that TIVA may be associated with improved long-term survival outcomes compared with volatile anesthesia in patients undergoing surgery for esophagogastric malignancies, particularly among patients undergoing gastrectomy. However, the available evidence is largely retrospective and heterogeneous, limiting causal interpretation. Further prospective multicenter randomized studies are required to clarify the oncological impact of anesthetic techniques in upper gastrointestinal cancer surgery. 

## Supplementary Information

Below is the link to the electronic supplementary material.

Supplementary Material 1

Supplementary Material 2

Supplementary Material 3

Supplementary Material 4

Supplementary Material 5

Supplementary Material 6

Supplementary Material 7

Supplementary Material 8

Supplementary Material 9

Supplementary Material 10

Supplementary Material 11

## Data Availability

No datasets were generated or analysed during the current study.
